# Exploring the feasibility, sustainability and the benefits of the GrACE + GAIT exercise programme in the residential aged care setting

**DOI:** 10.7717/peerj.6973

**Published:** 2019-06-04

**Authors:** Samantha Fien, Tim Henwood, Mike Climstein, Evelyne Rathbone, Justin W.L. Keogh

**Affiliations:** 1 School of Health, Medical and Applied Sciences, CQUniversity, Mackay, Queensland, Australia; 2 Health Science and Medicine, Bond University, Robina, Queensland, Australia; 3 Southern Cross Care, North Plympton, South Australia, Australia; 4 School of Health and Human Sciences, Southern Cross University, Gold Coast, Queensland, Australia; 5 Water Based Research Unit, Bond University, Robina, Queensland, Australia; 6 Physical Activity, Lifestyle, Ageing and Wellbeing Faculty Research Group, University of Sydney, Sydney, New South Wales, Australia; 7 Human Potential Centre, Auckland University of Technology, Auckland, New Zealand; 8 Kasturba Medical College, Mangalore, Manipal Academy of Higher Education, Manipal, Karnataka, India

**Keywords:** Ageing, Walking, Geriatrics, Resistance training

## Abstract

**Background:**

The feasibility and benefits of a 24-week targeted progressive supervised resistance and weight-bearing exercise programme (Group Aged Care Exercise + GAIT (GrACE + GAIT)) in the residential aged care (RAC) setting was investigated as very little peer-reviewed research has been conducted in relation to exercise programmes of this duration in this cohort.

**Methods:**

A quasi-experimental study design consisting of two groups (control and exercise) explored a 24-week targeted progressive supervised resistance and weight-bearing exercise programme (GrACE + GAIT) in two RAC facilities in Northern New South Wales, Australia. A total of 42 adults consented to participate from a total of 68 eligible residents (61.7%). The primary outcome measures were feasibility and sustainability of the exercise programme via intervention uptake, session adherence, attrition, acceptability and adverse events. Secondary measures included gait speed and the spatio-temporal parameters of gait, handgrip muscle strength and sit to stand performance.

**Results:**

Twenty-three residents participated in the exercise intervention (mean (SD) 85.4 (8.1) years, 15 females) and 19 in the control group (87.4 (6.6) years 13 females). Exercise adherence was 79.3%, with 65% of exercise participants attending ≥70% of the sessions; 100% of those originally enrolled completed the programme and strongly agreed with the programme acceptability. Zero exercise-related adverse events were reported. ANCOVA results indicated that post-intervention gait speed significantly increased (*p* < 0.001) with an 18.8% increase in gait speed (m/s).

**Discussion:**

The GrACE + GAIT programme was shown to be feasible and significantly improve adults living in RAC facilities gait speed, handgrip strength and sit to stand performance. These results suggest that the GrACE + GAIT programme is suitable for use in the RAC sector and that it has the potential to reduce disability and improve function and quality of life of the residents.

## Introduction

On average one million adults use Australia’s government funded aged care services on a typical day, with the majority (71%) of these adults living in residential aged care (RAC) facilities (Australian Institute of Health and Welfare, 2016). Older adults residing in RAC often have multiple chronic diseases, a sarcopenic status and take multiple prescribed medications (Australian Institute Health and Welfare, 2013; Fien et al., in press; Sluggett et al., 2017). These factors often place the residents at a high risk of adverse events (Australian Institute Health and Welfare, 2013). Ironically, adults living in RAC are among the least researched older adult group even though they have the highest rates for falls, hospital admissions and are among the highest consumers for prescribed medications (Australian Institute Health and Welfare, 2013). Residing in a RAC facility can be associated with a reduction in physical activity and mobility (i.e., physical performance) (Cichocki et al., 2015; Reid et al., 2013). In fact, older adults, especially RAC adults, require a lifestyle with regular exercise to reduce their risk of disease and increase their quality of life (Hassan et al., 2016; Tkatch et al., 2016).

Sarcopenia is defined by the European Working Group on Sarcopenia in Older People (EWGSOP2) as “low muscle strength, low muscle quantity or quality and low physical performance” (Cruz-Jentoft et al., 2019) (Table 1). The physical decline of gait speed in RAC adults is known to reflect a range of age-related adverse processes such as disability, cognitive impairment, falls, mortality, institutionalisation and hospitalisation (Abellan van Kan et al., 2009; Allali et al., 2017; Cruz-Jentoft et al., 2019; Keogh et al., 2015).

**Table 1:** Exercise intervention GrACE + GAIT (Phase I and Phase II).

**Variable**	**Intervention: Phase I**		**Intervention: Phase II**	
	Description	Equipment	Description	Equipment
**Duration**	12-weeks (0–12-weeks)		12-weeks (13–24-weeks)	
**Number of sessions per week**	45 min twice weekly		45 min twice weekly	
**Conditioning phase**	Included		Not included	
**Weight-bearing and resistance exercises**	Chair stands, chair dips, calf raises and hip flexor/abdominal lifts, trunk twists, and bicep curl and shoulder press.	Dumbbells (0.5, 1, 1.5 & 2 kg)	Chair stands, chair dips, calf raises and hip flexor/abdominal lifts, trunk twists, and bicep curl and shoulder press.	Dumbbells (0.5, 1, 1.5 & 2 kg)
	2 sets of 8–10 repetitions	Chairs	3 sets of 10–12 repetitions	Chairs
	Progressions in weights occurred every two weeks unless contraindicated due to medical or physical conditions		Progressions in weights occurred every two weeks unless contraindicated due to medical or physical conditions	
**Gait exercises**	No gait exercises		Progressions in the following exercises occurred every alternate two weeks to weight exercises, unless due to medical or physical conditions: Heel and toe raises, stepping in different directions, step-ups, and task-specific balance work for unstable base of support	Exercise balls
**Instructor**	Primary investigator (two days/week)		Primary investigator one day/week	
			RAC staff (physiotherapists) one day/week	

Gait speed is a common tool used to diagnose the muscle function component of sarcopenia and is clinically relevant to older adults (Abellan van Kan et al., 2009; Peel, Kuys & Klein, 2013; Studenski et al., 2011; Guralnik et al., 2000). The threshold for older adults to be considered as having normal habitual gait speeds is ≥0.8 m/s which was reported from a meta-analysis study containing 2,888 adults living in RAC (Kuys et al., 2014). This same study reported a mean habitual gait speed of 0.48 m/s (95% confidence interval (CI) 0.40–0.55 m/s) for adults living in RAC (Kuys et al., 2014). Work by our research team involving 102 randomly selected adults living in RAC reported a mean ± SD gait speed of 0.37 ± 0.26 m/s (Keogh et al., 2015). This dangerously low gait speed and its association with many adverse events supports the need for the development and assessment of novel interventions that can be translated to the RAC setting to counter the age-related decline in physical performance (Abellan van Kan et al., 2009; Auyeung et al., 2014; Ten Brinke et al.,  2018).

While well supported by evidence to improve overall physical wellbeing and mobility, progressive resistance and weight-bearing exercise is not commonly available in RAC facilities. This may reflect the lack of understanding of what constitutes feasible, safe, sustainable and effective longer-term exercise participation. An inefficient translation of research principles (time, duration and frequency) to practice may also be a factor to consider (Fien et al., 2016; Sievanen et al., 2014; Valenzuela, 2012). Progressive resistance training has also been found to improve gait speed, muscle strength and physical performance in the RAC setting (Chou, Hwang & Wu, 2012; Reid et al., 2017; Valenzuela, 2012). Although the magnitude of changes in physical performance such as gait speed is typically substantially less than that of the strength improvement (Hortobagyi et al., 2015). This suggests that these exercise programmes may need to target spatio-temporal parameters, in particular, stride length, step time and support base as these three parameters predicted 89% of the variance in gait speed in adults living in RAC (Fien et al., in press).

Progressive resistance training offers numerous benefits beyond improvements in muscle strength alone for older adults, particularly those living in RAC setting (Papa, Dong & Hassan, 2017; McPhee et al., 2016). Several reports have demonstrated improvements in balance, functional mobility, stability limits, and fall prevention (Bossers et al., 2014; Chou, Hwang & Wu, 2012; Papa, Dong & Hassan, 2017). Resistance training can reduce the effect of age-related changes in muscle function and improve performance of activities of daily living requiring postural stability and gait speed (Fien et al., 2016; McPhee et al., 2016; Papa, Dong & Hassan, 2017). It has also been demonstrated that these significant increases in functional performance with resistance training can be achieved even at the age of 90 years old (Papa, Dong & Hassan, 2017). However, there is currently limited support for the feasibility and sustainability of longer-term exercise programmes (Fien et al., 2016; Seitz et al., 2012; Valenzuela, 2012; Wesson et al., 2013).

The aim of this study was to evaluate the feasibility and benefits of a targeted supervised exercise programme for adults living in RAC. The primary focus was on investigating longer-term feasibility and obtaining a greater understanding of the mechanisms underlying the potential gait speed improvements than what was done previously with a shorter, more generalised pilot exercise programme in this setting (Fien et al., 2016). Data such as this, may have major practical applications to improving mobility and reducing disability in residential aged care.

## Methods and Material

**Figure 1:**
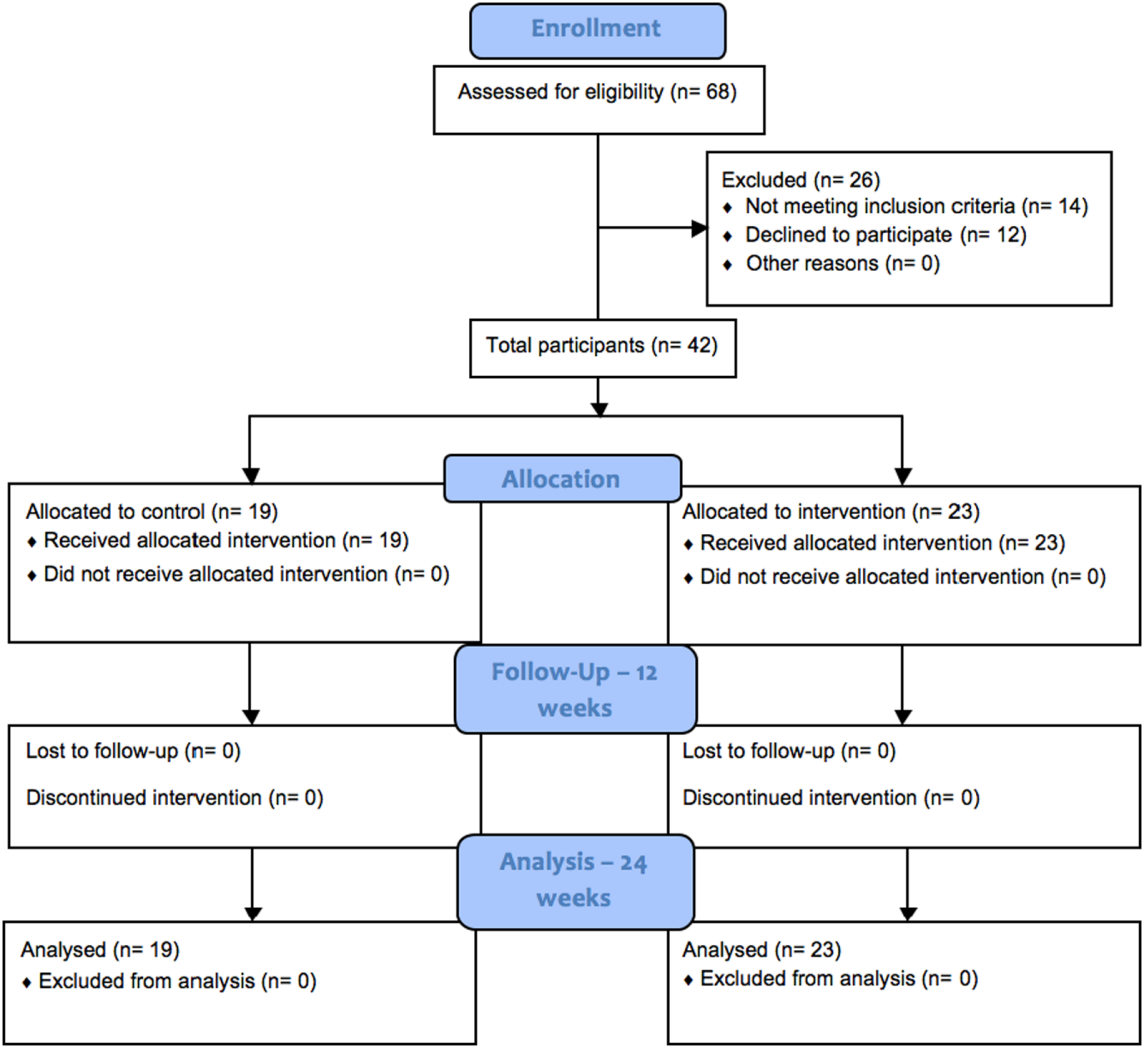
CONSORT flow diagram of recruitment and assessment of study participants. .

A quasi-experimental study design was used that involved two Australian RAC facilities. An overview of recruitment and delivery is given in Fig. 1. A total of 26 out of 68 participants eligible for enrolment discontinued on to the next stage with 14 not meeting the inclusion criteria and 12 declining to participate. For this feasibility study, sample size calculation was not carried out; the sample size was limited due to the restrictions around the eligibility and availability of participants in the RAC setting.

Participants were eligible if they were:

i. Aged over 65 years,
ii. Residing in a RAC for longer than three weeks,
iii. Able to walk with a walker and/or walking stick or could self-ambulate for the test (including those who have had knee and hip replacements) and,
iv. Could provide informed consent (self- or by proxy).

The exclusion criteria included:

i. End-stage terminal and/or life expectancy <6-months (ethical reasons),
ii. Two-person transfer or unable to self-ambulate (due to increased falls risk),
iii. Unable to communicate or follow instructions and,
iv. Dangerous behaviours endangering the client or research staff.

Following a discussion and an information sheet about the study the participants provided their informed consent if they wished to participate. A total of 42 participants took part in the study, with the primary investigator responsible for observing and administering all of the assessments. There were no withdrawals from either group during the study. In brief, participants were identified as eligible and capable of participating by the RAC Service Manager and the Registered Nurses. Residents were then allocated into either the usual care or the exercise group based on the following variables: the physical environment of the RAC facilities (multi-storey buildings, furniture, safety and location of the rooms, RAC facilities reduced staffing and staff available to transport residents to and from their rooms to exercise class and the complexity of the RAC setting). The exercise programme took place over a six-month period from June to December 2016. Ethical approval to conduct this study was attained from Bond University’s Human Ethics Research Committee (RO 1823), with the trial protocol published at Clinical Trial Registry (ID NCT02766738). For blinding purposes, the list of eligible participants was given to the primary investigator (from the RAC staff and management) to eliminate bias when selecting participants. Whilst first data analysis was completed by the statistician within the research team rather than the primary investigator to rule out bias.

### GrACE + GAIT exercise intervention

The progressive exercise intervention “GrACE + GAIT” consisted of a 24-week supervised training programme that was divided into two phases, each consisting of 12-weeks. These phases are detailed in Table 1. The first phase involved an identical training programme to that previously been reported in a pilot study (Fien et al., 2016). The sample of adults living in RAC in this study differed to those reported in the previous study as they were recruited from a Northern NSW region from June 2017. The second phase of the exercise programme in the current study was developed based on findings from an observational study involving 100 RAC residents, in which three spatio-temporal parameters (stride length, step time and support base) were found to predict 89% of the variance in gait speed (Fien et al., in press). It was identified that there was a need for a greater proportion of standing rather than seated exercises and targeted spatio-temporal gait parameters to enhance mobility.

Phase I of the exercise programme commenced with a four-week conditioning period, allowing participants the opportunity to achieve correct technique, minimise potential for adverse events and adapt to the training protocol (Fien et al., 2016). In the subsequent eight-week period, sets, repetitions and resistance were progressively increased as participants increased their exercise tolerance. The second phase did not have a four-week conditioning period (see Table 1). In terms of the progression of the exercises, weight progression occurred every two weeks (unless due to medical or physical conditions) and this occurred in both phases. In Phase II, changes in weights lifted and in the gait exercises occurred every alternate two weeks. The gait exercises included heel and toe raises, stepping in different directions, step-ups, and task specific balance work for unstable base of support.

### Usual care group

All participants assigned to the usual care group continued their regular activities during the intervention period.

### Data collection

All measures in this study have been previously validated for use with older adults, and their protocols reported elsewhere (Cruz-Jentoft et al., 2010; Fien et al., 2016; Henwood, Wooding & De Souza, 2013; Sterke et al., 2012). Pre- and post-assessments were completed one-on-one by the primary investigator in the residents’ room except for the gait speed test. Due to the space required (minimum 7.66 m in a straight line), gait speed was assessed in a communal room. The primary investigator also supervised the exercise sessions whereby RAC staff or volunteers monitored or were able to join in on the exercise sessions with the participants.

### Participant demographics

Residents were assessed for sarcopenia, cognitive status, body mass index (BMI), body fat percentage (%) and lean mass at baseline. The anthropometric data collected allowed for each resident to be screened for sarcopenia according to the EWGSOP guidelines (Cruz-Jentoft et al., 2010). The EWGSOP screening algorithm consisted of assessing gait speed, handgrip strength and muscle mass (i.e., lean mass). In order to be defined sarcopenic, individuals needed to have below normal levels of muscle mass and muscle strength or performance, using gender-specific cut-off points (Cruz-Jentoft et al., 2010). A simple five-item questionnaire, the “SARC-F” was also used to subjectively diagnose sarcopenia, i.e., 0–2 points for each component with 0 = best to 10 = worst (Cruz-Jentoft et al., 2019; Malmstrom & Morley, 2013) whereby a participant scoring 4 or more is described as “predictive of sarcopenia and poor outcomes”. The total number of mobility aids were also recorded with the category of either ambulant or mobility aids (including: walking stick, walking frame, wheelie walker and wheelchair). Cognitive status was quantified via the Mini Cog (Borson et al., 2000), with a total score of 1 or 2 out of 5 indicating a higher likelihood of dementia classified for this test as cognitive impairment. Body fat and lean mass were measured using bioelectrical impedance analysis (BIA, Maltron BF-906 body fat analyser, Essex, UK) (Chien, Huang & Wu, 2008; Senior et al., 2015).

Chronic conditions were defined as functional limitations that are not manifestations of physical disease but permanent or long-standing problems such as developmental disorders, limb dysfunction and visual impairment (Goodman et al., 2013). Prescribed medications were classified as medication requiring a doctor’s prescription or being supplied by an authorised health care professional as deemed by the Therapeutic Goods Administration with the Department of Health Australian Government (Australian Government, 2019).

### Primary outcome measure

#### (i) Feasibility measurements

Feasibility was evaluated against recruitment rate, session adherence, attrition, acceptability and adverse events (Bower et al., 2014; Fien et al., 2016; Peddle-McIntyre et al., 2012; Suttanon et al., 2013). Recruitment rate was defined as the proportion of residents who consented to participate from the group deemed eligible (Peddle-McIntyre et al., 2012). Session adherence was measured as the percentage of sessions attended out of 48 sessions offered (Bossers et al., 2014; Bower et al., 2014; Fien et al., 2016; Henwood, Wooding & De Souza, 2013). Attrition was defined by the proportion of people who dropped out of the intervention (Mullen et al., 2013). Acceptability was measured via a satisfaction survey involving a series of questions with a five-point Likert scale (Peddle-McIntyre et al., 2012). This was completed post-training to assess the burden of participation and testing, as well as overall trial satisfaction (Peddle-McIntyre et al., 2012). Adverse events (within the exercise group) were defined as incidents in which harm or damage occurred to the participant, including but not limited to, falls and fall-related injuries, musculoskeletal or cardiovascular incidents and problems with medication and medical devices (Australian Government, 2014) as a result of their involvement in the exercise programme. These adverse events were recorded by the primary investigator via observation and questioning the nursing staff and the residents’ pre, during and post exercise sessions.

#### (ii) Sustainability measurement

Sustainability was defined as “the existence of structures and processes that allow a programme to leverage resources to effectively implement and maintain evidence-based policies and activities” (Schell et al., 2013, pg. 2). Sustainability is critical to RAC facilities, if a programme is not sustainable and delivered by the appropriate staff member/s, it can be a waste of time, money and resources. This may limit the ability of the organisation’s ability to achieve RAC goals and effectively implement exercise programmes (Goodman & Steckler, 1989; Schell et al., 2013). In regards to the training of an internal staff member to implement an exercise programme, research reveals the sustainability of an exercise programme within the context of community-dwelling adults is increased if locally trained instructors are utilised (Estabrooks et al., 2011; Stewart et al., 2006). Sustainability was assessed on whether the exercise programme could be completed with one exercise instructor for a group of up to 10 adults living in RAC in a session. The class was performed in a communal multi-purpose room and that it only required 1 set of dumbbells (ranging in weight: 0.5 kg–2.0 kg), an exercise ball and a dining room chair per resident.

### Secondary outcome measures

#### (i) Gait speed

The data of the resident’s gait speed and spatio-temporal parameters were collected using the Gait Mat II system (Manufacturer: EQInc; Model: Gait Mat II). A description of how this device has been used previously in RAC facilities described in an earlier paper (Fien et al., in press). Briefly, the measurement of gait speed and the spatio-temporal parameters commenced from a walking start with a two metre (m) wooden platform on either sides of the GaitMat II (3.66 m (11.91 ft) long) to reduce the effect acceleration and deceleration may have on gait speed (Kressig et al., 2004). Participants performed three trials of this walking task and the average gait speed (m/s) was used for data analysis.

#### (iii) Spatio-temporal parameters

The spatio-temporal parameters were collected from the GaitMat II (Walsh) and included the following measurements: step length (m), stride length (m), support base (m), step time (s), swing time (s), stance time (s), single support time (s) and double support time (s) and are defined in Table 2, which has been included from a previous paper from the research team (Fien et al., in press).

#### (iv) Handgrip strength

Isometric handgrip strength was measured with participants seated in a chair and their elbow bent to 90° at their side. Participants were instructed to squeeze a handgrip dynamometer (Sammons Preston Roylan, Bolingbrook, IL, USA) as hard as they could (maximum ability) in their dominant hand for a period of up to five seconds (Mathiowetz, 2002). The best result of two trials was recorded for analysis (Roberts et al., 2011).

**Table 2:** Spatio-temportal gait determinants and definitions as defined by the GaitMat II manual (Walsh., n.d.).

**Spatio-temporal gait determinants**	**Definitions**
Step length	The distance from the first switch closure of one footprint to that of the footprint on the contralateral side.
Stride length	The distance from the first switch closure of one footprint to the next footprint on the ipsilateral side.
Support base	The medial lateral distance across the mat to the innermost switch closure for one footprint from the innermost switch closure of the previous footprint on the contralateral side.
Step time	The time to the earliest switch closure of a footfall from the earliest switch closure of the previous footfall on the contralateral side.
Swing time	The time to the earliest switch closure of a footfall from the latest switch opening of the previous footfall on the ipsilateral side.
Stance time	The time to the latest switch opening of a footfall from the earliest switch closure of the same footfall.
Single support time	The time to the earliest switch closure of the next footfall on the contralateral side from the latest switch opening of the previous footfall on the contralateral side.
Double support time	The time to the latest switch opening of the previous footfall on the contralateral side from the earliest switch closure of a footfall.

#### (v) Sit to stand performance

Sit to stand performance was measured from sitting on a chair. Participants stood to a full standing position from the chair as many times as possible in 30 s whilst keeping their arms crossed against their chest. Due to the fatiguing nature of this test, only one trial was performed (Millor et al., 2013).

### Statistical analysis

Descriptive statistics were produced on the baseline characteristics with all continuous variables presented as either mean ± SD or median (range) depending on the statistical distribution of the variables. Categorical variables were summarized as counts and percentages (%). In circumstances where participants were unable to complete a physical measure, (*n* = 8 for sit to stand performance), they were given the lowest score, generally zero. Baseline characteristics of the two groups were compared using either independent *t*-tests or Mann–Whitney *U* tests for continuous variables, and chi-square analysis for categorical variables.

The main inferential test undertaken was a one-way repeated measures analysis of covariance (ANCOVA) to assess the effect of the exercise intervention, after adjusting for potential between-group baseline differences in gait speed and the number of chronic diseases. The actual score at 24-weeks was used as the dependent variable and the intervention group was used as the main predictor whilst the baseline score and chronic diseases were used as covariates. Assumptions for the ANCOVA were checked. Statistical significance was set at *p* ≤ 0.05 *a priori*. Further analyses were performed using paired *t*-tests to investigate if there was a significant pre-post-test difference in the variables between time points within each group (baseline to 12-weeks, 12-24-weeks and baseline to 24-weeks). A Bonferroni correction was used for the paired *t*-tests due to multiple comparisons so that *p* < 0.008 was the reference for statistical significance. Statistical Package for the Social Sciences (SPSS, version 20, IBM, Armonk, NY, USA) was used for data analysis.

## Results

Forty-two of the 68 residents (61.7%) deemed eligible provided informed consent to participate. The total cohort had a mean ± SD age of 86.3 ± 6.7 years (range 66–97 years, 28 females), gait speed of 0.61 ± 0.17 m/s, BMI of 26.6 ± 3.7 kg/m^2^, number of chronic diseases 9.4 ± 2.4, number of prescribed medications 10.9 ± 4.3 and 66.7% were cognitively impaired (see Table 3). There were no significant between group differences at baseline except for chronic diseases (*p* = 0.03).

**Table 3:** Baseline characteristics of the residents (*N* = 42) in the RAC setting.

**Characteristic**	**Control (*n* = 19)**	**Exercise intervention** (*n* = 23)	**Total group (*N* = 42)**
Females, *n* (%)	13 (68.4%)	15 (65.2%)	28 (66.7%)
Age (yrs)	87.4 (4.5)	85.4 (8.1)	86.3 (6.7)
BMI (kg/m^2^)	26.6 (3.7)	26.4 (3.8)	26.5 (3.6)
Body Fat Mass (%)	36.6 (9.0)	37.5 (8.9)	37.0 (8.9)
Fat Mass (kg)	23.7 (7.6)	24.5 (8.2)	24.1 (7.9)
Lean Mass (kg)	41.0 (8.4)	40.0 (7.0)	40.5 (7.8)
Handgrip strength, kg (Median, range)	11 (6.0–18.4)	13 (6.0–32.0)	12 (6.0-32.0)
Sit to stand assistance, *n* (%)	13 (68.4%)	10 (43.5%)	23 (54.8%)
Sit to stand, *n*	1 (0.75)	2 (1.7)	1.5 (1.4)
Mobility aids (Aid used), *n* (%)	15 (78.9%)	16 (69.6%)	31 (73.8%)
Mini COG, impaired, *n* (%)	14 (73.7%)	14 (60.9%)	28 (66.7%)
Medical Conditions, #	10.3 (2.3)^*^	8.7 (2.3)	9.4 (2.4)
Medications, #	11.6 (3.3)	10.3 (4.9)	10.9 (4.3)
EWGSOP Sarcopenic status, count	0	0	0
SARC-F total score (out of 10)	4 (1.6)	4 (1.9)	4 (1.3)
Gait speed, m/s	0.58 (0.19)	0.64 (0.16)	0.61 (0.17)
Step length, m	0.43 (0.05)	0.41 (0.07)	0.42 (0.06)
Stride length, m (Median, range)	0.88 (0.44–1.08)	0.81 (0.44–1.15)	0.84 (0.44–1.15)
Support base, m (Median, range)	0.15 (0.07–0.40)	0.16 (0.09–0.31)	0.15 (0.07–0.40)
Step time, s (Median, range)	0.63 (0.34–1.00)	0.62 (0.34–1.11)	0.63 (0.34–1.11)
Swing time, s (Median, range)	0.42 (0.33–0.62)	0.40 (0.33–0.68)	0.42 (0.33–0.68)
Stance time, s (Median, range)	0.87 (0.54–1.46)	0.85 (0.68–1.50)	0.86 (0.54–1.50)
Single support time, s (Median, range)	0.42 (0.33–0.79)	0.40 (0.22–0.79)	0.41 (0.22–0.79)
Double support time, s (Median, range)	0.25 (0.19–0.41)	0.21 (0.15–0.41)	0.22 (0.15–0.41)

**Notes.**

Data is presented as Mean Standard Deviation unless otherwise specified.

BMI Body Mass Index EWGSOP European Working Group Sarcopenia in Older People kg kilogram m metre mini COG mini cognitive test *n* number s second SARC-F sarcopenia questionnaire yrs years # number % percentage

* Significant difference between Control and Exercise Intervention groups (was only found in the number of medical conditions).

A total of 42 residents from the eligible 68 participated in the study. Overall adherence rate was 79.3%, while no participants attended all 48 sessions. Adherence to the exercise sessions was quite good with 15 residents (65%) attending 34 or more exercise sessions (70%). The most common reasons for non-attendance included family/friends visiting, medical appointments/surgeries, sickness and organised external activities. There were no exercise-related adverse events or dropouts during the programme, with all 42 commencing participants completing the post-programme assessment. All participants agreed or strongly agreed that they enjoyed the programme, that they would be happy to continue, were physically better than prior to commencing the programme and benefited from participation (refer to Appendix 1).

The ANCOVA on gait speed was used to detect any difference between the two groups whilst controlling for potential baseline differences in gait speed and the number of chronic diseases. The model explained 66.8% of the variation in gait speed (see Table 3). The results indicate that there was an 18.8% change in gait speed for the exercise group from pre (0.64 m/s) to post (0.76 m/s) intervention. There was also a large treatment effect size on gait speed at the end of the 24-week intervention (*F* (1,38) = 15.6, *p* < 0.001, partial *η*^2^ = 0.29). The eta-squared *η*^2^ (proportion of the total variance attributed to an effect) was found to be of a large effect size for the variables: baseline gait speed and exercise group in Table 4.

**Table 4:** Analysis of covariance (ANCOVA) model of a 24-week GrACE + GAIT intervention in 42 residents.

**Variables**	**Beta coefficient (95% CI)**	***p*-value**	Partial Eta Squared (*η*^2^)^a^
**Baseline gait speed (m/s)**	0.66 (0.43, 0.89)	<0.001^*^	0.462
**Baseline chronic diseases (*n*)**	−0.024 (−0.05, −0.003)	0.024^*^	0.128
**Exercise group GrACE + GAIT**^b^	0.15 (0.07, 0.23)	<0.001^*^	0.290

**Notes.**

* Statistically significant *p* < 0.05.

CI Confidence Interval m/s metres per second *n* number

a Cohens effect size^2^: small = 0.01, medium = 0.059, large = 0.138.

b The results for the exercise group are displayed since the reference category was the control group.

Paired *t*-tests were performed on the following variables: gait speed, gait spatio-temporal parameters, handgrip strength and sit to stand performance to describe the pre-post-test changes in mean ± SD within each of the two groups.

### Gait speed

**Figure 2:**
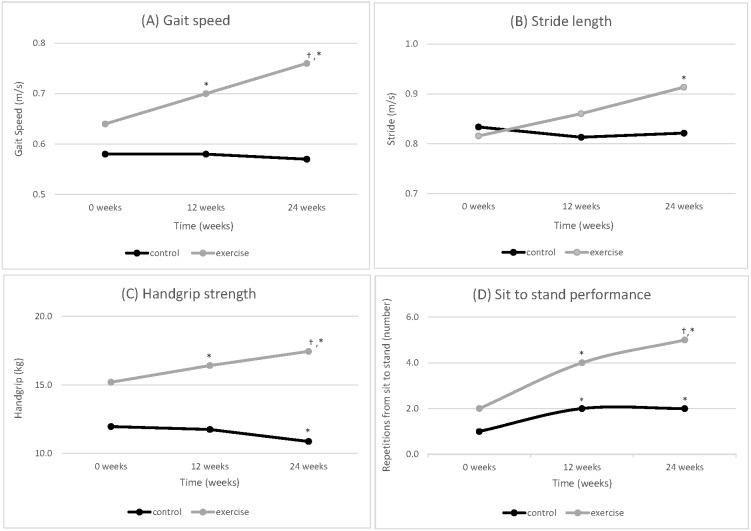
Mean physical performance (A) Gait speed, (B) Stride length, (C) Handgrip strength and (D) Sit to stand performance-control (*n* = 25) versus exercise group (*n* = 23) over the 24-week GrACE + GAIT exercise programme (*N* = 48). Note: *, difference from baseline 0 weeks to 12 weeks or 24 weeks that is statistically significant within group (*P* < 0.008); †, difference between 12 weeks to 24 weeks that is statistically significant within group (*P* < 0.008).

During the 24-week intervention, the usual care group’s gait speed remained unchanged (0.58 ± 0.19 m/s to 0.57 ± 0.18 m/s, *p* = 0.35), whilst the exercise group significantly increased from 0.64 ± 0.16 m/s to 0.76 ± 0.18 m/s (*p* = 0.002). There were no significant improvements in the usual care group in the first phase (*p* = 0.75) and the second phase (*p* = 0.44) Whilst gait speed significantly improved in the exercise group in the first phase (*p* = 0.001) and in the second phase (*p* = 0.04) (Fig. 2A).

### Gait spatio-temporal parameters

During the 24-week intervention, the usual care group’s stride length remained unchanged (0.83 ± 0.16 m to 0.82 ± 0.18 m, *p* = 0.39), whilst the exercise group significantly increased from 0.81 ± 0.16 m to 0.91 ± 0.15 m (*p* = 0.007). These improvements in stride length for the exercise group (*p* = 0.007) after 24-weeks were not significant during the first (*p* = 0.07) or second phase (*p* = 0.07) (Fig. 2B). None of the other gait spatio-temporal parameters were significantly altered at any time point for either group during the 24-week intervention or during the first or second phase of the intervention.

### Handgrip strength

During the 24-week intervention, the usual care group’s handgrip strength significantly decreased (12.0 ± 3.7 kg to 10.9 ± 3.9 kg, *p* < 0.001), whilst the exercise group significantly increased (15.2 ± 7.2 kg to 17.4 ± 8.5 kg, *p* = 0.001). The usual care group did not have significant difference in the first phase (*p* = 0.35) but found significance in the second phase (*p* = 0.004). Whilst handgrip strength significantly improved in the exercise group in the first phase (*p* = 0.001) and in the second phase (*p* = 0.04) (Fig. 2C).

### Sit to stand performance

During the 24-week intervention, the usual care group’s sit to stand performance significantly increased from 1.0 ± 0.7 to 2.0 ± 1.5 sit to stands (*p* = 0.003). The exercise group significantly improved their sit to stand performance from 2.0 ± 1.7 to 5.0 ± 2.2 sit to stands (*p* < 0.001) over the 24 weeks. Whilst the usual care group had a significant improvement in the first phase (*p* = 0.003), there was no significant difference in the second phase (*p* = 0.23). Sit to stand performance significantly improved in the exercise group in both the first (*p* = 0.003) and second phases (*p* = 0.001) (Fig. 2D).

## Discussion

This study sought to determine the feasibility and sustainability of a longer duration (24-weeks) progressive resistance-training programme than what has been typically performed in previous studies (6–12 weeks) of adults living in RAC (Fien et al., 2016; Sievanen et al., 2014). For the 23 residents who were enrolled in the exercise programme, total session adherence rate was 79.3%. This attendance rate appeared similar to or greater than reported in previous exercise studies involving adults living in RAC with average age ranging from 82 to 86 years old (Alvarez-Barbosa et al., 2014; Bossers et al., 2014; Fien et al., 2016; Sievanen et al., 2014; Toots et al., 2016). There were no dropouts or exercise-related adverse events reported in the exercise group during the 24-week programme, reiterating that the exercise programme was feasible for this extended period of time. This study also indicated that the older adults who were in the exercise intervention (GrACE + GAIT) twice a week for 24-weeks was feasible when living in RAC and also significantly improved their gait speed.

Further evidence supporting the sustainability of the GrACE + GAIT was that it utilized one exercise instructor for a group of up to 10 adults living in RAC in a session (Lazowski et al., 1999). The exercise class was performed in a communal multi-purpose room and that it only required 1 set of dumbbells (ranging in weight: 0.5 kg–2.0 kg) an exercise ball and a dining room chair per resident. In regard to sustainability, the programme was able to maintain two exercise classes per week for 24-weeks. During the second phase, the primary investigator conducted one class per week, whilst an internal RAC staff member conducted the second exercise class. Such findings provide some indication of long-term sustainability of the programme, as it would appear to be financially more viable and easily delivered by exercise professionals and/or trained RAC staff than other exercise interventions examined in the RAC context (Alvarez-Barbosa et al., 2014; Auyeung et al., 2014; Bossers et al., 2014; Estabrooks et al., 2011; Hassan et al., 2016; Henwood et al., 2015; Sievanen et al., 2014; Stewart et al., 2006; Timonen et al., 2002). A Cochrane Systematic review in 2013 evaluated 67 trials involving 6,300 participants within RAC facilities and concluded that physical rehabilitation may be effective. However, there was insufficient evidence to determine if improvements were sustainable or which interventions where most appropriate (Crocker et al., 2013). The findings of the current study therefore provide some relatively strong indication for how effective progressive resistance and weight-bearing training programmes may be implemented over the longer term in RAC facilities.

However, it must be acknowledged that although the GrACE programme was found to be feasible for those who participated in this study, this amounted to only ∼32% of the population of the RAC facility. Collectively, the results of this study suggest that further feasibility trials may need to target adults living in RAC who were ineligible for this study (Gibbs et al., 2015) and also examine some of the issues influencing recruitment rates from those who were eligible to participate (Kalinowski et al., 2012).

The exercise group also experienced a significant increase in gait speed of 0.12 m/s, whereas the usual care group decreased their mean gait speed by 0.02 m/s. The training related improvements in gait speed for the exercise group were almost double that of the 0.07 m/s (CI [0.02–0.11] m/s) increase reported in a meta-analysis by (Chou, Hwang & Wu, 2012) and provide important evidence that exercise can counter the expected annual decline in gait speed of 0.03–0.05 m/s per year for older adults (Auyeung et al., 2014; Onder et al., 2002). Further, the increase of 0.12 m/s reported in the study is greater than the 0.10 m/s increase in gait speed that has been considered a meaningful change in previous studies involving older adults (Chui, Hood & Klima, 2012; Fritz & Lusardia, 2009).

Based on previous research that demonstrated 89% of the variance in the adults living in RAC gait speed was predicted by stride length, step time and support base (Fien et al., in press). Therefore, it was not surprising that stride length was also significantly improved after the 24-week period in the exercise group. However, no significant increases in stride length were observed at 12-weeks. An increase in stride length would typically require improved balance and/or increased anterior-posterior impulse production (Sterke et al., 2012; Taylor et al., 2013). Thus, the lack of significant increase in stride length in the first 12-weeks may have reflected the majority of exercises being performed in a seated position during this time. The inclusion of the gait-focused standing exercises in the second phase of the programme may have been crucial to optimise improvements in gait speed and spatio-temporal parameters in adults living in RAC. In the context of community-dwelling older adults research, frail status adults, compared to non-frail status, have a reduced gait speed and stride length with increased stride time (Cesari et al., 2005; Montero-Odasso et al., 2005). Gait speed has been reported as one of the strongest predictors of adverse outcomes, such as disability, falls, or hospitalization in a range of older populations (Cesari et al., 2005; Purser et al., 2006; Schwenk et al., 2014). However, assessment of gait speed does not, in and of itself, provide insight into the specific gait pattern, which in turn may limit the sensitivity and specificity of discrimination between different frailty and health statuses (Schwenk et al., 2014).

On this basis, we would recommend that exercise prescriptions for older adults in RAC might need to be longer than 12-weeks duration and have a progression of general (seated) to more specific balance and weight-bearing strengthening exercises to improve gait speed without increasing the risk of exercise-related adverse events like falls. The requirement for exercise programmes exceeding 12-weeks may reflect the multifactorial processes and multiple sensorimotor systems that influence gait speed (Brach & VanSwearingen, 2013; Jimenez-Garcia et al., 2019; Pedersen & Saltin, 2015).

### Study limitations

The primary limitation was the lack of a randomized control trial (RCT) design and the lead researcher not being blinded to participant allocation. This lack of an RCT design and researcher blinding, which partially reflected the challenges of conducting exercise trials in the RAC setting, may reduce the internal validity of the study’s findings. However, there were no statistically significant differences at baseline between groups for all the participants’ characteristics apart from chronic diseases which was a covariate accounted for, in our analysis of covariance. We acknowledge that 42 participants assessed represented a recruitment rate of only 52% of the participants initially thought to be eligible for the study and only about 32% of the facility’s population. However, the potential for research of bias was reduced in that the primary outcome of gait speed was assessed by the GaitMat II system which does not allow any researcher bias to influence the results recorded. It is also acknowledged that 3D biomechanical analyses would have also provided additional information regarding the potential mechanisms underlying the improvement in gait speed and stride length, such as the potential for improved postural stability and greater anterior posterior impulse production during the stance phase.

## Conclusions

This study found the 24-week exercise programme was feasible and sustainable in the RAC setting and the resident’s gait speed and physical performance improved in the GrACE + GAIT exercise group at the conclusion of the intervention. In order to ensure such exercise programmes, become a part of usual care in RAC, additional RCT designs need to examine how exercise. In particular, progressive resistance and weight-bearing training may improve a range of other indices of health and well-being and whether such programmes are economically viable.

##  Supplemental Information

10.7717/peerj.6973/supp-1 Supplemental Information 1 Raw data Raw data file for data analyses and table/figure preparation for the feasibility, sustainability and benefits of an exercise programme in residential aged care setting.

10.7717/peerj.6973/supp-2 Supplemental Information 2 CONSORT checklist

## Appendices

1. Exercise group participant questionnaire post 24-week completion of the GrACE + GAIT exercise programme.

**Table:**

**Question**					
Did you have any concern(s) with the GrACE programme *before starting* exercise programme?					
	SA = 0	A = 2	U = 4	D = 10	SD = 7
Did you have any concern(s) with the GrACE + GAIT programme *upon completing* the exercise programme?					
	SA = 0	A = 0	U = 2	D = 18	SD = 3
Did you enjoy participating in the GrACE + GAIT programme?					
	SA = 23	A = 0	U = 0	D = 0	SD = 0
Do you believe the GrACE programme was well organised?					
	SA = 20	A = 3	U = 0	D = 0	SD = 0
Did you find the GrACE programme impede on your daily routine?					
	SA = 0	A = 1	U = 3	D = 16	SD = 3
Would you be happy to continue participating in the GrACE + GAIT programme or something similar?					
	SA = 23	A = 0	U = 0	D = 0	SD = 0
Overall, would you rate your current physical condition to be better than before you started the GrACE + GAIT programme?					
	SA = 23	A = 0	U = 0	D = 0	SD = 0
SA, Strongly agree; A, Agree; U, Unsure; D, Disagree; SD, Strongly disagree					

2. Feedback from the open-ended questions in the participant post GrACE + GAIT exercise programme.

**Table:**

**Feedback questions**	**Comments**
What was the best aspect of the GrACE + GAIT exercise programme?	“Everybody being in a group together”
	“I enjoyed the class”
	“I liked the instructor”
	“Getting out of my room”
	“Being able to do something”
	“Having the energy to do it”
	“The exercise balls”
	“I was able to push myself”
	“Seeing everyone”
	“Having morning tea with everyone afterwards”
	“I liked the class”
	“Walking across the plank”
	“Improving”
What aspect of the GrACE + GAIT needs improvement?	“More scheduled time for exercises”
	“I wish we didn’t get interrupted in our exercise class by other people”
	“More space”
	“Nothing”
	“None”
	“Needs to go for longer”
Other comments/feedback	“Wonderful”
	“Thank you for your time”
	“Thank you”
	“Fantastic”
	“Very organised”

## Additional Information and Declarations

### Data Availability

The following information was supplied regarding data availability: The raw measurements are available in Supplemental File.

### Clinical Trial Registration

The following information was supplied regarding Clinical Trial registration: The trial protocol is published at Clinical Trial Registry (ID NCT02766738).
https://clinicaltrials.gov/ct2/show/NCT02766738?term=NCT02766738&rank=1.
